# What the Country Is Doing

**Published:** 1914-08-15

**Authors:** 


### A STATE OF WAR.

### The Country and the Care of the Sick and Wounded.

### WHAT THE COUNTRY IS DOING.

THE war has come upon us so suddenly that it has not been possible to publish complete information of what is being done throughout the country with regard to the provision of new and further hospital accommodation. This week our reports are mainly confined to the chief centres. They will necessarily be suggestive rather than complete, for the work itself is not finished. We have tried to arrange them in such an order as to make it easy to follow on the map each of the divisions dealt with.

### Hospital Managers and Officials, Please Help.

**We shall be grateful to hospital managers and officials throughout the United Kingdom who will kindly take the trouble to ascertain as far as possible exactly what has been done in their district in these matters, and to send us as complete an account as possible, accompanied by newspaper cuttings and any other documents or papers which bear on the subject, for which we shall be glad to make adequate acknowledgment. We should like to have a correspondent in every centre of population and in every important county division, especially on the East Coasts of England and Scotland. We therefore invite every hospital official and hospital manager who would like to undertake the duties of a correspondent to communicate with the Editor of "The Hospital," 28 Southampton Street, Strand, W.C., without delay.**

### VOLUNTARY AID DETACHMENTS.

It is a good thing that subsequent to the South African War a system of Voluntary Aid Detachments should have been organised, with the knowledge and co-operation of the authorities, under the principal auspices of the British Red Cross Society. These organisations are centralised in county units, and are worked through Executive Committees in conjunction with general meetings consisting of the vice-presidents, honorary secretaries, and chief officials. In times of peace those who have joined these organisations have been instructed in first aid, and have had systematic instruction and drills in the work which will devolve upon them in regard to ambulance service, cooking, rest stations, hospitals, and many other useful matters. It will be their duty to form rest stations and transport trains in their own localities, and to be prepared, if requested by the War Office, to form a hospital or hospitals to supply the needs of each county. The Women's Voluntary Aid Detachments have been very active, and many of them, as we have reason to know from our inquiries during recent inspections of the hospitals, have made themselves remarkably efficient. All honour to them. The accounts which have so far been published of the meetings of the executive and other authorities seem to point to the absence of practical knowledge in regard to the cost of equipping and establishing a hospital for, say, 100 beds. At a recent meeting at Ipswich it was stated that the executive committee had decided to make a direct offer to the War Office to equip a hospital for 100 beds, able to go and serve wherever and whenever it might be required. It was pointed out, however, that Ipswich should not at the present time form hospitals until they received a direct order. The executive, it is stated, have issued an appeal for £3,000 to equip a hospital for field service, but if it is to contain 100 beds this sum may not be anything like enough.

### A CIVIL DIRECTOR-GENERAL.

Again, the *Aberdeen Daily Journal* points out that naval doctors and members of the Royal Army Medical Corps know quite well what they have to do, but they cannot be sure how much help they may rely upon from civilians. This absence of knowledge is a very important factor in the situation, and the suggestion made to Lord Kitchener in our leading article this week to appoint a Civil Director-General, with authority to organise and equip the whole of the new hospital units supplied by civilians for the care of the sick and wounded throughout the country, would speedily remove this condition of uncertainty. It is essential to know the exact position, both in regard to the number of hospitals and beds as well as in regard to the funds which the public will supply.

In considering the best kind of organisation and equipment to meet the object in view, it must be borne in mind that the position of naval doctors and of the sick and wounded sailors differs materially from that of members of the R.A.M. Corps and of the disabled soldiers. Until the hour of action the sailor is engaged in his ordinary work and in the enjoyment of sound health. After an action the treatment required is almost entirely surgical. Immediately after a naval battle the desideratum will be to remove the wounded without a moment's avoidable delay to the nearest port and to place them in a hospital there. To fulfil the Navy requirements, one of the first essentials will therefore be to have an adequately equipped and sufficiently large hospital ready in every port, to each of which must be attached an able surgical staff. The amount of hospital accommodation required by the Navy must therefore depend upon the amount of actual fighting done and the results of the actions fought. In the case of the soldier circumstances differ materially. The camping and progress of an army through a country, under the best conditions, cannot be free from inhygienic accompaniments. The life of the soldier, especially at the commencement of a campaign, and during the preliminary organisation and drills attendant upon life in camps, must inevitably lead to a relatively large sick list, which will call for the services of a number of physicians, or medical practitioners, as distinct from the surgeons. The surgeons may have little or nothing to do unless there is actual fighting, but the medical practitioners will be kept constantly busy; and on their skill and devotion, combined with the completest hygienic efficiency, the health of the troops must largely depend. It follows that the hospital accommodation required for the Army will be in any event infinitely greater, if the campaign is to last, than we hope will in any circumstances be needed for the treatment of our sailors. Of course the latter accommodation may have to be increased because of the humane practice pursued by the English race in time of warfare, which entails that the wounded enemy shall have every care and attention directly we have provided for our own sick and wounded.

A consideration of the circumstances just explained will make it clear that so far as the new hospital units which will have to be provided up and down the country, and especially on the East Coast of Great Britain, for the accommodation of the sick, if it is to be adequate and thoroughly efficient, should be organised under conditions which will attract the maximum of public support and interest, and secure the very best results for the sick and wounded at a minimum cost. That is why we have urged the essential importance at the outset of the appointment of a Civil Director-General to take charge of the organisation and equipment of the chain of new hospitals, convalescent and kindred establishments which are about to be set up throughout the country.

### Hospitals at the Ports.

The port hospitals for our sailors are quite a distinct Department. They must be placed under conditions which will render the maximum of facilities for the speedy removal of the wounded from the vessels which bring them to the port hospital for immediate treatment. These hospitals might, and we expect will, be largely arranged by a reconstruction of some of the big warehouses which most of the docks in each port contain, for in this way efficiency, economy, expedition, and the minimum of suffering and risk to our sailors on landing should be secured. At Hull, however, arrangements have been made to equip several Temporary hospitals from which the wounded sailors would be sent to the Base hospitals which will be situated further inland.

